# Shakuyaku-Kanzo-To Prevents Angiotensin Ⅱ-Induced Cardiac Hypertrophy in Neonatal Rat Ventricular Myocytes

**DOI:** 10.7759/cureus.74064

**Published:** 2024-11-20

**Authors:** Hideaki Tagashira, Fumiha Abe, Ayako Sakai, Tomohiro Numata

**Affiliations:** 1 Department of Integrative Physiology, Akita University Graduate School of Medicine, Akita, JPN; 2 Department of Integrative Physiology, Graduate School of Medicine, Akita University, Akita, JPN

**Keywords:** cardiomyocyte hypertrophy, japanese kampo medicine, l-type ca2+ channel, ros, shakuyaku-kanzo-to

## Abstract

The global incidence of mortality due to heart failure (HF) is on the rise, presenting a significant challenge in various regions, including Japan. There is an urgent need for innovative prevention and treatment strategies to address this issue. Traditional medicine, particularly Japanese Kampo medicine (JKM), has been proposed as a potential therapeutic approach and has undergone examination in clinical trials related to HF. However, the deficiency of robust scientific evidence underscores the necessity for further exploration into the cardioprotective mechanisms of JKM. This study systematically examines the cardioprotective effects of Shakuyaku-kanzo-to (SKT), a specific JKM with limited application in cardiac care. Utilizing neonatal rat ventricular myocytes, we assessed the direct effects of SKT on myocardial hypertrophy. Methodologies included immunohistochemistry for cell size and a plate reader for quantifying cell survival, intracellular calcium levels ([Ca^2+^]_i_), and reactive oxygen species (ROS) production. In addition, quantitative reverse transcription polymerase chain reaction (RT-PCR) was employed for gene expression analysis. The findings reveal that SKT significantly mitigates angiotensin Ⅱ (AngⅡ)-induced cardiomyocyte hypertrophy and cell death, while also reducing elevated [Ca^2+^]_i _and ROS production associated with this condition. Furthermore, co-administration of nifedipine, an L-type Ca^2+^ channel (L-Ca^2+^) blocker, demonstrated that SKT antagonizes L-Ca^2+^ actions. These results indicate that SKT offers protection against AngⅡ-induced cardiomyocyte hypertrophy by inhibiting L-Ca^2+^-mediated pathways. Consequently, this research highlights the potential of SKT as a promising therapeutic agent for cardiac applications, paving the way for new preventive and treatment strategies for HF.

## Introduction

Cardiovascular diseases, such as myocardial hypertrophy and heart failure (HF), pose significant challenges to global health, characterized by considerable morbidity and mortality rates [1]. Among the various factors contributing to cardiovascular pathologies, dysregulation of intracellular calcium (Ca^2+^) signaling and excessive production of reactive oxygen species (ROS) are recognized as critical mediators of cardiomyocyte hypertrophy and injury [2,3].

Among this dysregulation, angiotensin Ⅱ (AngⅡ) is a crucial element of the renin-angiotensin system that plays a significant role in the induction of cardiomyocyte hypertrophy and cell death. This occurs through its effects on intracellular Ca^2+^ homeostasis and the production of ROS. In light of the advancements in pharmacological therapies, there has been an increasing interest in investigating the therapeutic potential of traditional herbal medicines, particularly regarding their ability to alleviate cardiovascular diseases [4,5]. In particular, Shakuyaku-kanzo-to (SKT), a traditional Japanese Kampo medicine, has been traditionally used to treat pain and muscle cramps [6,7]. Nonetheless, the specific effects of SKT on cardiac health, particularly regarding cardiomyocyte hypertrophy and cell death, have yet to be thoroughly explored. Given its prevalent use and potential therapeutic advantages, there is significant interest in investigating the cardioprotective properties of SKT.

In this study, we aimed to elucidate the effects of SKT on AngⅡ-induced cardiomyocyte hypertrophy and injury, focusing on its regulatory actions on intracellular Ca^2+^ signaling and ROS production. By employing neonatal rat ventricular myocytes (NRVMs) as an experimental model, we investigated the direct effects of SKT on cardiomyocytes and explored its underlying mechanisms of action.

Our findings provide valuable insights into the potential therapeutic utility of SKT in preventing AngⅡ-induced cardiomyocyte hypertrophy and cell death, thereby offering novel strategies for managing cardiovascular diseases. In addition, elucidating the mechanistic basis of SKT's cardioprotective effects may pave the way for developing targeted therapeutic interventions for cardiovascular pathologies.

## Materials and methods

Materials

The Japanese Kampo medicine SKT (TJ-68) was obtained from Tsumura & Co. (Tokyo, Japan). The reagent was dissolved in dimethyl sulfoxide (DMSO) at a concentration of 250 mg/ml and subsequently diluted to the desired final concentration in aliquots. AngⅡ was purchased from Peptide Institute Inc. (Osaka, Japan). Nifedipine (NIF) was purchased from Sigma-Aldrich (MO, USA). Rhodamine-conjugated phalloidin was acquired from Abcam (ab235138, Abcam, Cambridge, UK). The ROS indicator, H_2_DCFDA, was obtained from Invitrogen (Thermo Fisher Scientific, MA, USA), while the Ca^2+^ indicator, Fluo-4, was obtained from Dojindo Laboratories (Kumamoto, Japan). All other chemicals used in this study were guaranteed to be of reagent-grade quality, obtained from Nacalai Tesque, Inc. (Kyoto, Japan), Fujifilm Wako Pure Chemical Corp. (Osaka, Japan), and Sigma-Aldrich Co., LLC (St. Louis, MO, USA).

Animals

All animal experiments were conducted in strict adherence to the Guidelines for Care and Use of Laboratory Animals. Approval was obtained from the Animal Ethics Committee of Akita University, Japan, for a study period spanning from May 26, 2022, to May 25, 2025. The protocols were assigned ethics review numbers a-1-0412 and b-1-0408. Neonatal rats for the experiments were obtained from adult female Wistar rats (Japan SLC, Inc., Hamamatsu, Japan).

Cell culture

NRVMs were isolated from the hearts of Wistar rats aged one to three days. The rat pups were euthanized by decapitation by ethical guidelines. NRVMs were isolated using the Pierce Cardiomyocyte Isolation Kit (Thermo Fisher Scientific) following the provided protocol, as previously described [8]. Dispersed cells were cultured in Dulbecco's Modified Eagle Medium (DMEM; Sigma, D6429) supplemented with 10% fetal bovine serum (FBS) and penicillin/streptomycin (Nacalai Tesque, Kyoto, Japan) in a CO₂ incubator at 37°C.

Morphological analysis of NRVMs

The morphological analysis of NRVMs was conducted to assess the effects of 100 nM AngⅡ on cardiac hypertrophy, following established methodologies [8,9]. Cells fixed in 4% formaldehyde were stained with rhodamine-phalloidin (Abcam). The cross-sectional area (CSA) of the cells was quantified using fluorescence microscopy in conjunction with ImageJ software [10]. The 50% inhibitory concentration (IC_50_) and Hill slope of SKT were calculated using GraphPad Prism 9 software (GraphPad Software, CA, USA) through a sigmoidal dose-response equation.

Measurement of cell viability and cytotoxicity

Cell viability and cytotoxicity were assessed using a colorimetric MTT assay (Cell Counting Kit-8, Dojindo) and an LDH activity assay (Cytotoxicity LDH Assay Kit-WST, Dojindo), respectively. NRVMs were plated at a density of 4.0 × 10^5^ cells/mL in 96-well plates, cultured in DMEM, and treated with 100 nM AngII for 72 hours. Absorbance was measured at 450 nm for viability using a Multiskan JX plate reader (Thermo Fisher Scientific) and at 490 nm for cytotoxicity using an Infinite M200 microplate reader (Tecan Group Ltd.), with viability and cytotoxicity results presented as percentages relative to the control. Both assessments were conducted following previously established methodologies [8,11,12]. Cardiac cell hypertrophy was induced using 100 nM AngⅡ and cultured in 96-well plates. Briefly, SKT treatment was applied at a concentration of 500 μg/mL, as per the dosage used in previous studies on traditional herbal medicine, and was administered simultaneously with AngII [8].

Measurement of intracellular Ca^2+^ and ROS

Intracellular Ca^2+^ and ROS levels were measured in NRVMs following established protocols [8]. Cardiac cell hypertrophy was induced using 100 nM AngⅡ, and cells were subsequently cultured in 96-well plates. For the measurement of Ca^2+^, Fluo-4 AM (F312, Dojindo) was utilized, while the measurement of ROS was performed using H_2_DCFDA (C400, Invitrogen). Both fluorescent indicators were incubated with the cells for 30 minutes at 37°C in a CO_2_ incubator, and fluorescence intensity at excitation/emission wavelength = 485/538 nm was quantified using a fluorescence microplate reader (Fluoroskan Ascent, Thermo Fisher Scientific).

RNA isolation and real-time RT-PCR

RNA isolation was conducted following a previously described protocol [8,13]. Total RNA was extracted from NRVMs utilizing the NucleoSpin® RNA Plus kit (Takara-Bio, Shiga, Japan) in accordance with the manufacturer’s instructions. Reverse transcription of the total RNA samples was performed with the Prime-Script™ Ⅱ 1st strand cDNA Synthesis Kit (Takara-Bio). Quantitative real-time PCR was executed using the SYBR Green Real-Time PCR Master Mix-Plus (Toyobo Co., Ltd., Osaka, Japan) on a LightCycler® 480 System Ⅱ (Roche Diagnostics Ltd., Rotkreuz, Switzerland). Gene-specific primers were synthesized by Sigma-Aldrich (MO, USA). The sequence for the rat primers employed was as follows (Table 1).

**Table 1:** Primer sequences used in PCR ANP: atrial natriuretic peptide, BNP: brain natriuretic peptide, Ca_v_1.2: L-type Ca^2+^ channel main subunit α1C; β-actin, PCR: polymerase chain reaction

Gene	Forward primer	Reverse primer	GeneBank accession #	Size (bp)	Reference
ANP	5’-AAATCCCGTATACAGTGCGG-3’	5’-GGAGGCATGACCTCATCTTC-3’	NM_012612.2	105	[8]
BNP	5’-CCATCGCAGCTGCCTGGCCCATCACTTCTG-3’	5’-GACTGCGCCGATCCGGTC-3’	NM_031545.1	364	[8]
Cav1.2	5’-CATTGCCTCCGAACACTA-3’	5’-GAACTTTCCACCAAACAGC-3’	NM_012517.2	412	[14]
β-actin	5’-ACTATCGGCAATGAGCGGTTC-3’	5’-ATGCCACAGGATTCCATACCC-3’	NM_031144.3	77	[8]

Melting curve analysis was performed to affirm the specificity of the amplification. The relative expression levels of the target genes were normalized to the expression of the housekeeping gene β-actin using the ΔΔCt method. Furthermore, a representative gel image of the PCR products was captured using gel electrophoresis to confirm the specificity. PCR was conducted utilizing KOD-Plus-Ver.2 (Toyobo) as previously delineated [8]. The PCR products were separated on a 2% agarose gel and visualized using GelRedTM (Fujifilm Wako) staining.

Statistical analysis

Statistical analyses were performed utilizing GraphPad Prism software (version 9, GraphPad, San Diego, CA, USA). The results were presented as mean ± standard error of the mean (SEM). To evaluate the statistical significance between means, we applied Student's t-test following the verification of variance equality via an F-test. A p-value of less than 0.05 was deemed statistically significant. For multiple comparisons, one-way analysis of variance (ANOVA) was utilized, followed by Tukey's post-hoc test. To ensure the reproducibility of the results, the experiments were conducted a minimum of three times. The methodology for calculating the combination index (CI) was derived from a prior publication [15].

## Results

SKT inhibits AngⅡ-induced cardiomyocyte hypertrophy

AngⅡ is widely recognized for its capacity to induce cardiomyocyte hypertrophy [16]. In order to evaluate the impact of SKT on hypertrophy and its associated cellular damage, a concentration of 100 nM of AngⅡ was utilized [17,18].

**Figure 1:**
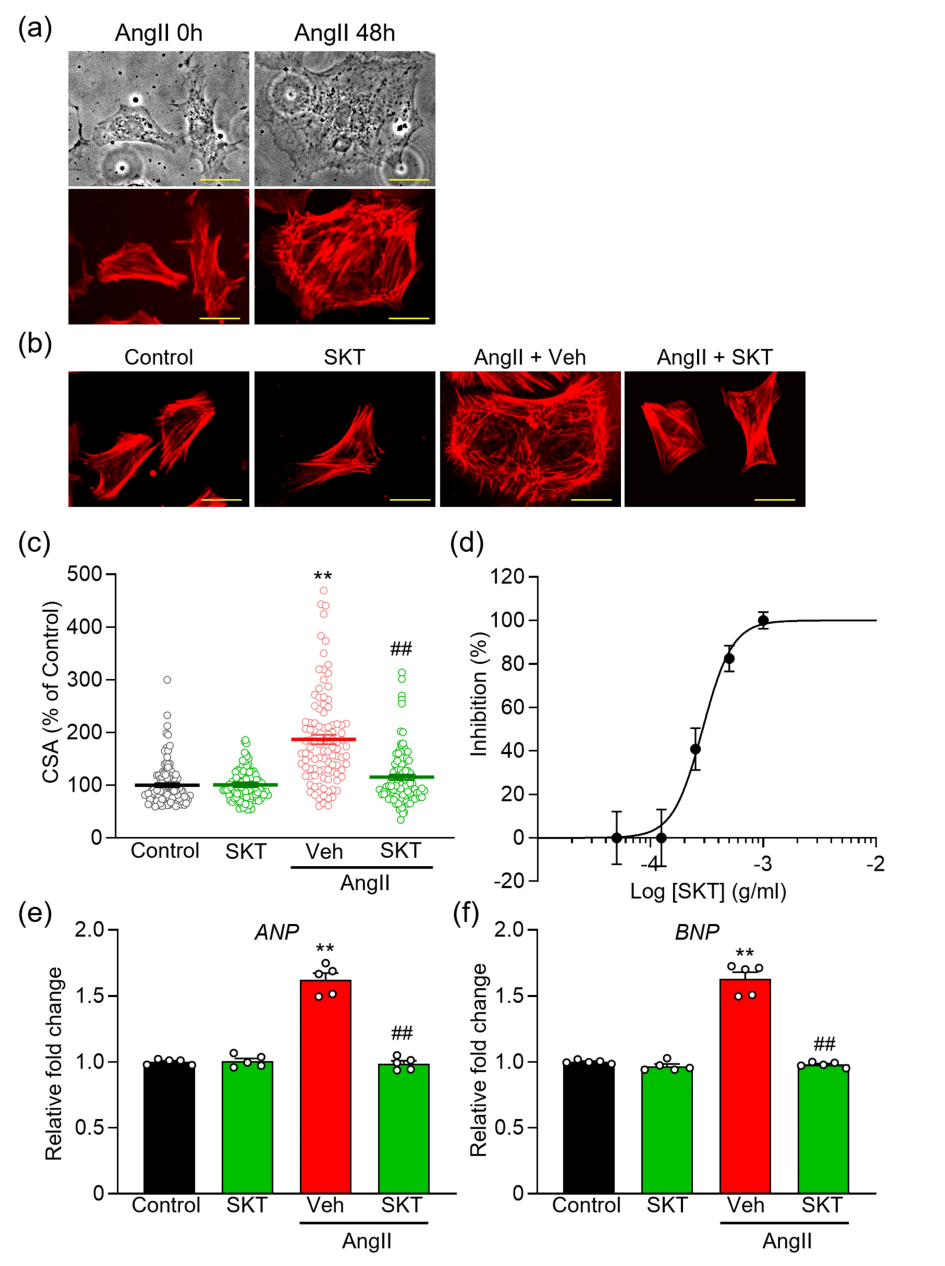
Effect of Shakuyaku-kanzo-to (SKT) on AngⅡ-induced cardiomyocyte hypertorophy (a) Representative cells are depicted in transmitted light images (top row) alongside rhodamine-conjugated phalloidin-stained fluorescence images (bottom row). NRVMs were characterized at 0 and 48 hours following the administration of AngⅡ. (b) Representative images of cells stained with rhodamine-conjugated phalloidin for each experimental group illustrate the effect of SKT (500 μg/ml) 48 hours after AngⅡ stimulation compared to the control group. (c) An evaluation of the antihypertrophic effect of SKT on AngⅡ-induced NRVM hypertrophy is presented through multiple cross-sectional area (CSA) measurements, as shown in panel (b). Each point represents one CSA measurement, and each group consists of >100 cells (n = 101-111). (d) Statistical analysis of the dose-dependent inhibition curve of SKT against AngⅡ-induced hypertrophy (n = 100-113). (e,f) Quantitative real-time PCR analysis of hypertrophic gene markers, ANP (e) and BNP (f) (n = 5). Each column represents the mean ± SEM. **, p < 0.01 indicates a significant difference compared to Control group. ##, p < 0.01 indicates a significant difference compared to AngⅡ + Veh group. Scale bar = 50 μm. SKT: Shakuyaku-kanzo-to, AngⅡ: angiotensin Ⅱ, NRVMs: neonatal rat ventricular myocytes, PCR: polymerase chain reaction, ANP: atrial natriuretic peptide, BNP: brain natriuretic peptide, SEM: standard error of the mean

After a 48-hour exposure to 100 nM AngⅡ, a significant increase in cardiomyocyte hypertrophy was observed (Figure 1a). The concentration of SKT applied in this study was 500 μg/ml, which was chosen based on literature indicating that concentrations of 100 μg/ml or higher are frequently employed in Kampo herbal medicine trials, with an effective range established between 250 and 500 μg/ml [13,19-28].

The treatment with SKT did not produce a significant change in the cross-sectional area (CSA) of cardiomyocytes when compared to the control group (Figures 1b, 1c: Control, SKT) However, it did significantly reduce AngⅡ-induced cardiomyocyte hypertrophy (Figures 1b, 1c: AngⅡ + Veh, AngⅡ + SKT). Furthermore, dose-dependent experiments demonstrated that SKT effectively inhibited AngⅡ-induced hypertrophy, exhibiting an IC_50_ value of 290.3 μg/ml and a Hill slope of 3.3 (Figure 1d).

To further clarify the impact of SKT on cardiac hypertrophy, we conducted quantitative reverse transcriptase polymerase chain reaction (qRT-PCR) experiments to assess the expression of key cardiac hypertrophic marker genes, specifically atrial natriuretic peptide (ANP) and brain natriuretic peptide (BNP). Our findings revealed a significant increase in the expression of these markers due to AngⅡ-induced cardiomyocyte hypertrophy. Notably, treatment with SKT resulted in a marked reduction in their expression levels (Figure 1e).

Protective effect of SKT against cell damage associated with AngⅡ-induced hypertrophy

**Figure 2:**
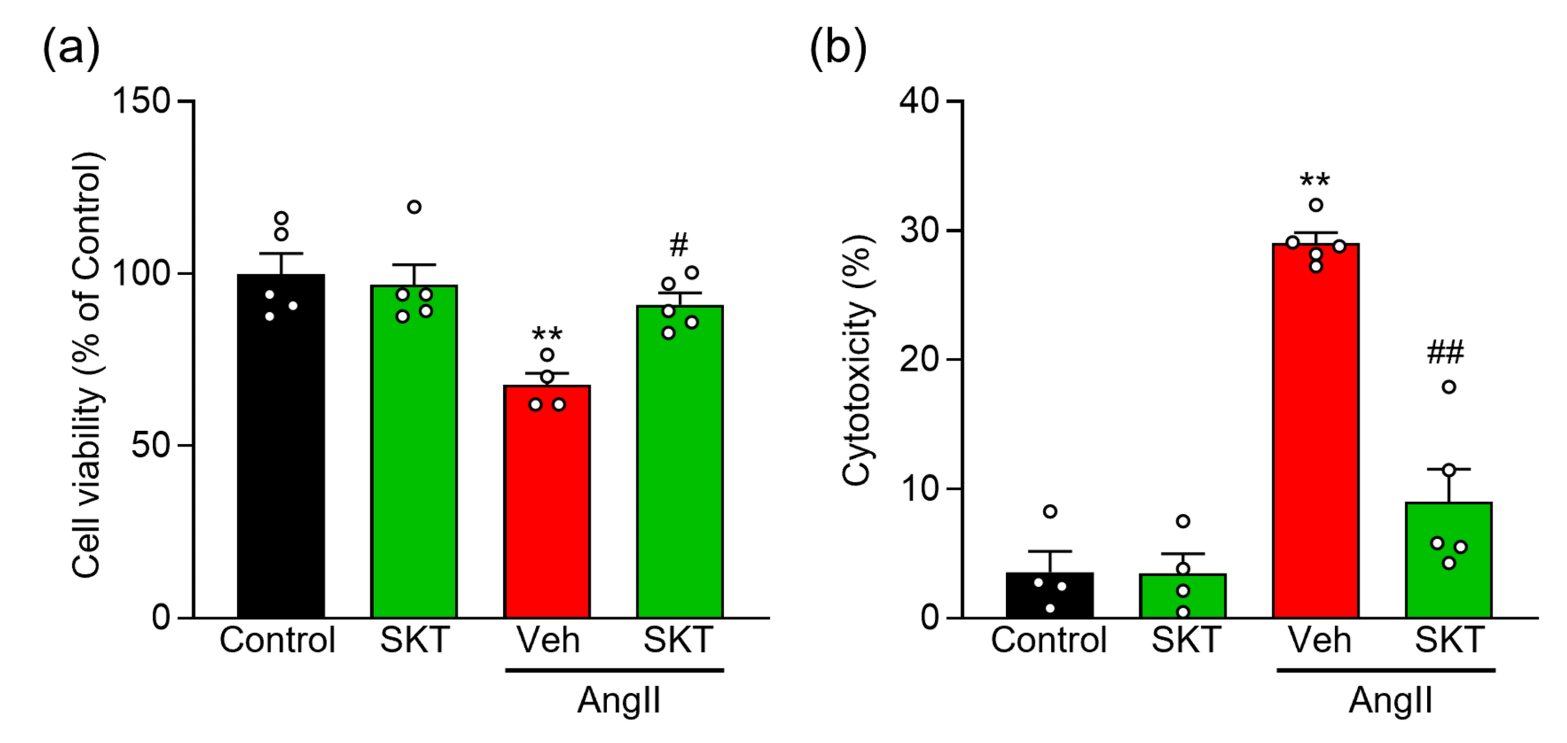
Effect of SKT on AngⅡ-induced NRVMs cell death Evaluation of the effect of SKT treatment on NRVMs after 48 h of AngⅡ administration. NRVM were treated with either vehicle (Control or Veh) or 500 μg/ml SKT (SKT) with or without AngⅡ induction. (a) The MTT assay assessed cell viability (n = 4-5). (b) Cytotoxicity was evaluated through LDH release assay (n = 4-5). Each column represents the mean ± SEM. **, p < 0.01 indicates a significant difference compared to the control group. #, p < 0.05 and ##, p < 0.01 indicate significant differences compared to the AngⅡ + Veh group. SKT: Shakuyaku-kanzo-to, AngⅡ: angiotensin Ⅱ, NRVMs: neonatal rat ventricular myocytes, SEM: standard error of the mean

Subsequently, we investigated the potential protective effects of SKT against cell damage linked to AngⅡ-induced hypertrophy. Our results demonstrated that SKT significantly mitigated the AngⅡ-induced reduction in cell viability and the corresponding increase in cytotoxicity (Figure 2a, 2b: AngⅡ + Veh, AngⅡ + SKT). Furthermore, it is noteworthy that treatment with 500 μg/ml SKT alone did not result in any significant alterations in cell viability or cardiomyocyte toxicity when compared to control cells (Figures 2a, 2b: Control, SKT).

SKT attenuates AngⅡ-induced abnormalities in intracellular Ca^2+^ and ROS levels

**Figure 3:**
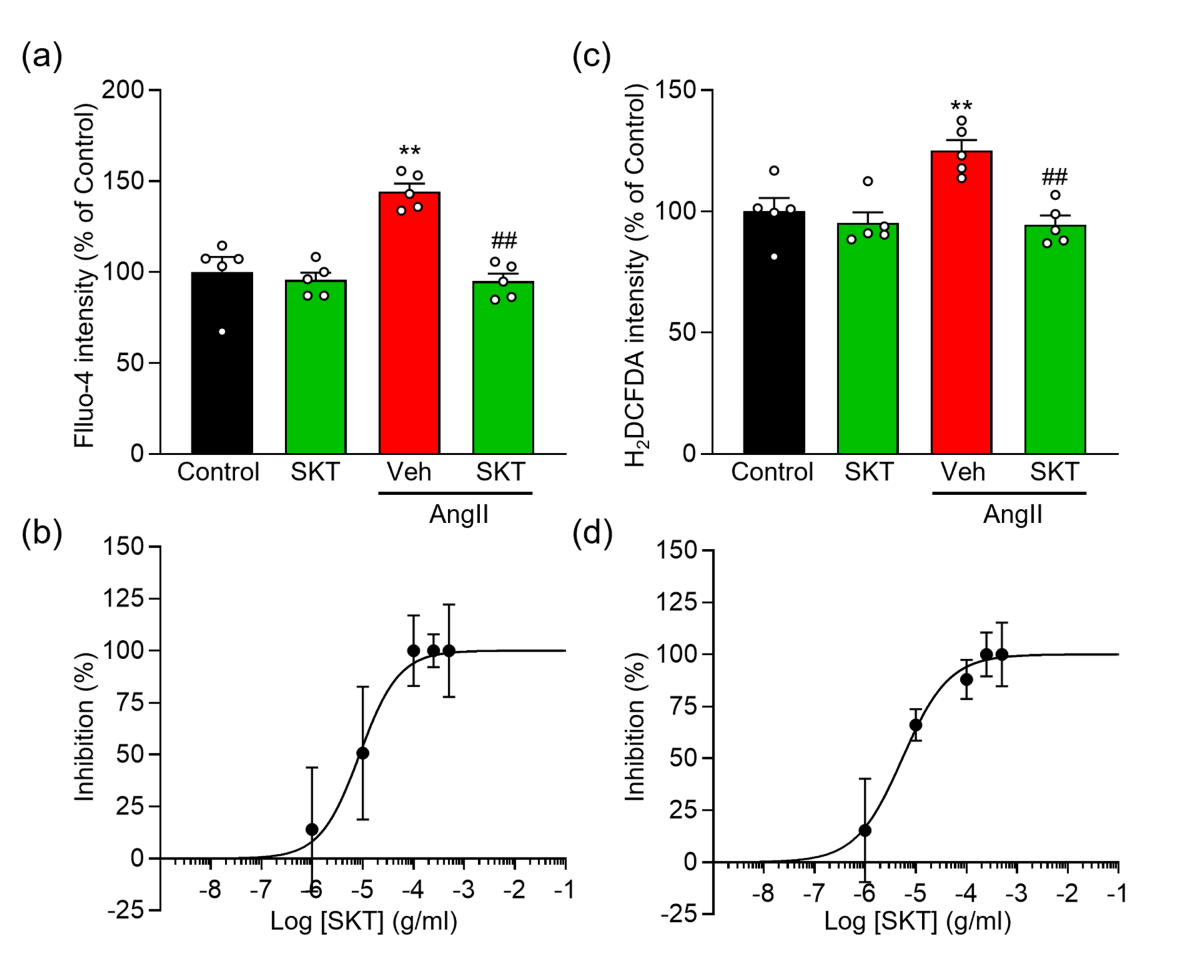
Effect of SKT on intracellular Ca2+ and ROS concentrations of AngⅡ-induced NRVMs Evaluation of the effect of SKT treatment on NRVM were treated with either vehicle (Control or Veh) or 500 μg/ml SKT (SKT) with or without AngⅡ induction. (a) Intracellular Ca^2+^ concentration was measured by Fluo-4 intensity (n = 5). (b) Statistical analysis of the dose-dependent inhibition curve of SKT against AngⅡ-induced intracellular Ca^2+^ elevation. (c) Intracellular ROS concentration was assessed through H_2_DCFDA intensity (n = 5). (d) Statistical analysis of the dose-dependent inhibition curve of SKT against AngⅡ-induced increase in ROS production. Each column represents the mean ± SEM. **, p < 0.01 indicates a significant difference compared to the control group. ##, p < 0.01 indicates a significant difference compared to the AngⅡ + Veh group. SKT: Shakuyaku-kanzo-to, ROS: reactive oxygen species, AngⅡ: angiotensin Ⅱ, NRVMs: neonatal rat ventricular myocytes, SEM: standard error of the mean

To investigate the myocardial protective mechanism of SKT, we examined the intracellular environment. It is widely recognized that cardiomyocyte hypertrophy and injury result from elevated intracellular Ca²⁺ and ROS levels [2,3]. Consequently, we sought to elucidate the cardioprotective effects of SKT by analyzing intracellular Ca²⁺ and ROS levels in NRVMs induced with AngⅡ. Consistent with previous studies [8,29,30], our findings indicate that treatment with AngⅡ for a duration of 48 hours resulted in a significant elevation in both intracellular Ca²⁺ and ROS levels (Figures 3a, 3c). By contrast, treatment with SKT markedly mitigated these AngⅡ-induced increases in Ca²⁺ and ROS. Specifically, the AngⅡ-induced elevation of intracellular Ca²⁺ was significantly diminished in the SKT treatment group in a dose-dependent manner, exhibiting an IC_50_ value of 8.6 μg/ml and a Hill slope of 1.1 (Figures 3a, 3b). Furthermore, ROS levels were similarly reduced in the SKT treatment group, demonstrating an IC_50_ of 5.4 μg/ml and a Hill slope of 0.9 (Figures 3c, 3d).

These results underscore the potential of SKT in conferring protection against AngⅡ-induced cellular disturbances.

SKT improves AngⅡ-induced cardiomyocyte hypertrophy by acting on antagonistic targets to Nifedipine

**Figure 4:**
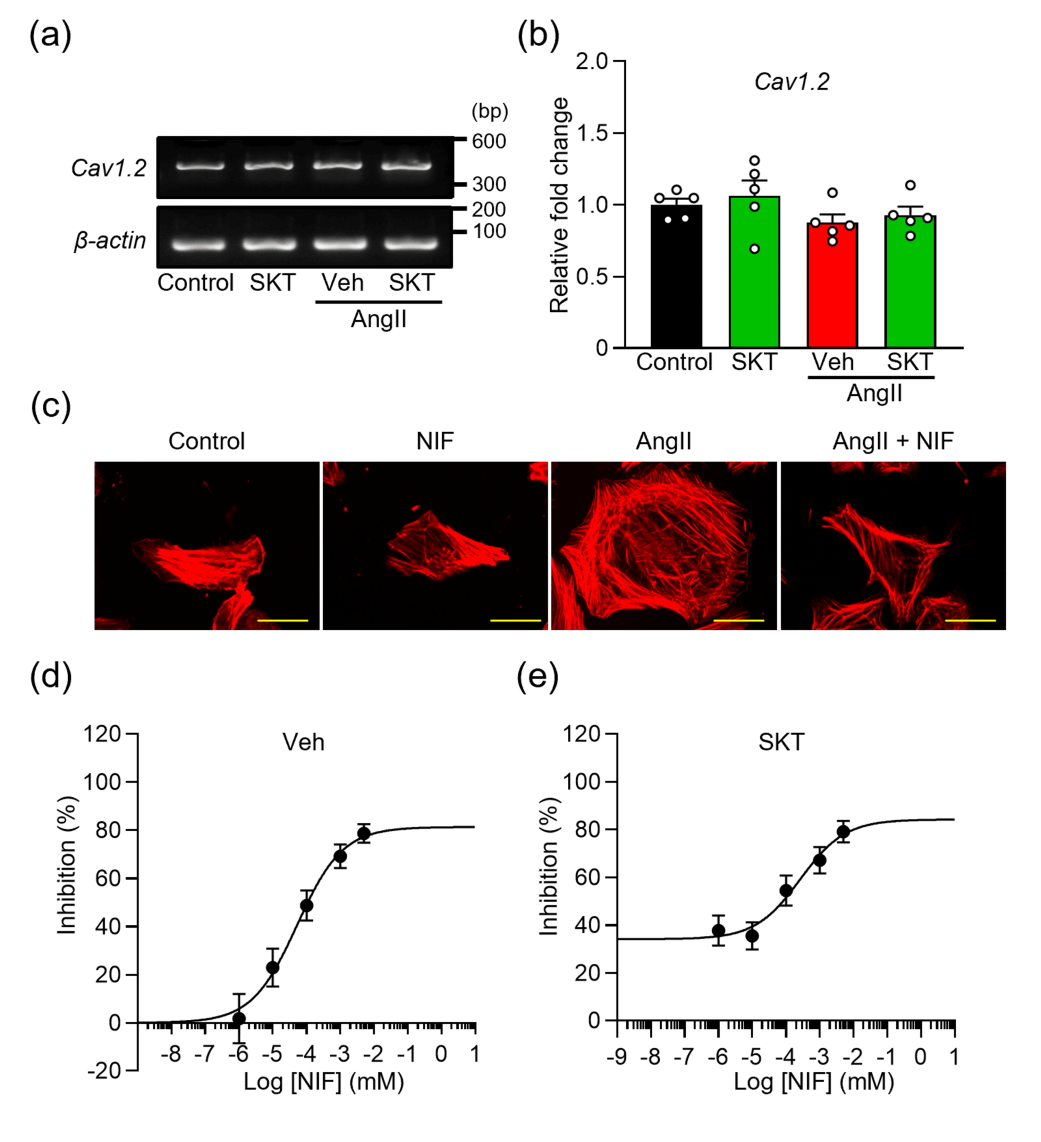
Effect of SKT on the L-type Ca2+ channel in AngⅡ-induced cardiomyocyte hypertrophy (a) mRNA expression of the CACNA1C gene encoding the L-type Ca^2+^ channel (Ca_v_1.2). PCR products obtained from NRVMs treated with vehicle (Control or Veh), or 250 μg/ml SKT, after 48 hours of AngⅡ-induced cardiomyocyte hypertrophy show the expression of Ca_v_1.2 and a constitutively transcribed control β-actin. (b) Quantitative real-time PCR analysis of Ca_v_1.2 mRNA (n = 5). (c) Representative images of cells stained with rhodamine-conjugated phalloidin to investigate the effect of NIF (1 μM) on AngⅡ-induced cardiomyocyte hypertrophy. (d) Dose-dependent inhibition curve of NIF on AngⅡ-induced cardiomyocyte hypertrophy (n = 103-119). (e) Dose-dependent inhibition curve of NIF combined with 250 μg/ml SKT on AngⅡ-induced cardiomyocyte hypertrophy (n = 100-109). Each group consisted of more than 100 cells. Each column represents the mean ± SEM. SKT: Shakuyaku-kanzo-to, AngⅡ: angiotensin Ⅱ, PCR: polymerase chain reaction, NRVMs: neonatal rat ventricular myocytes, SEM: standard error of the mean

It is well-established that the influx of Ca^2+^ through L-type Ca^2+^ channels (L-Ca^2+^) play a crucial role in AngⅡ-induced cardiomyocyte hypertrophy [31]. To investigate this further, we examined the expression of the *CACNA1C* gene (Ca_v_1.2), which encodes the primary α1C subunit responsible for forming L-Ca^2+^ in NRVMs. Results from RT-PCR indicated significant expression of Ca_v_1.2 in NRVMs. Notably, this expression remained relatively unchanged at the mRNA level in both AngⅡ-induced and control NRVMs, regardless of SKT treatment (Figures 4a, 4b).

To assess the impact of L-Ca^2+^ on AngⅡ-induced NRVMs, we measured the CSA using NIF, a selective blocker of L-Ca^2+^. The effects of NIF on cardiomyocyte hypertrophy exhibited a dose-dependent relationship, with an IC_50_ of 53.1 nM and a Hill slope of 0.6 (Figures 4c, 4d). Notably, 5 µM NIF was the maximum allowed due to cardiotoxicity observed at higher concentrations. These findings for the effects of NIF on NRVMs were consistent with previous studies involving cardiomyocytes [32-34].

Subsequently, we investigated to determine the combination index (CI) to elucidate the interaction of SKT with the NIF of the target. The concentration-dependent inhibition curve of NIF in the presence of 250 μg/ml SKT, which corresponds to approximately half the inhibitory effect of SKT calculated in Figure 1d, demonstrated a significant effect at NIF concentrations of 0.01 μM or higher, with a CI of 1.2 (see Figure 4e).

**Figure 5:**
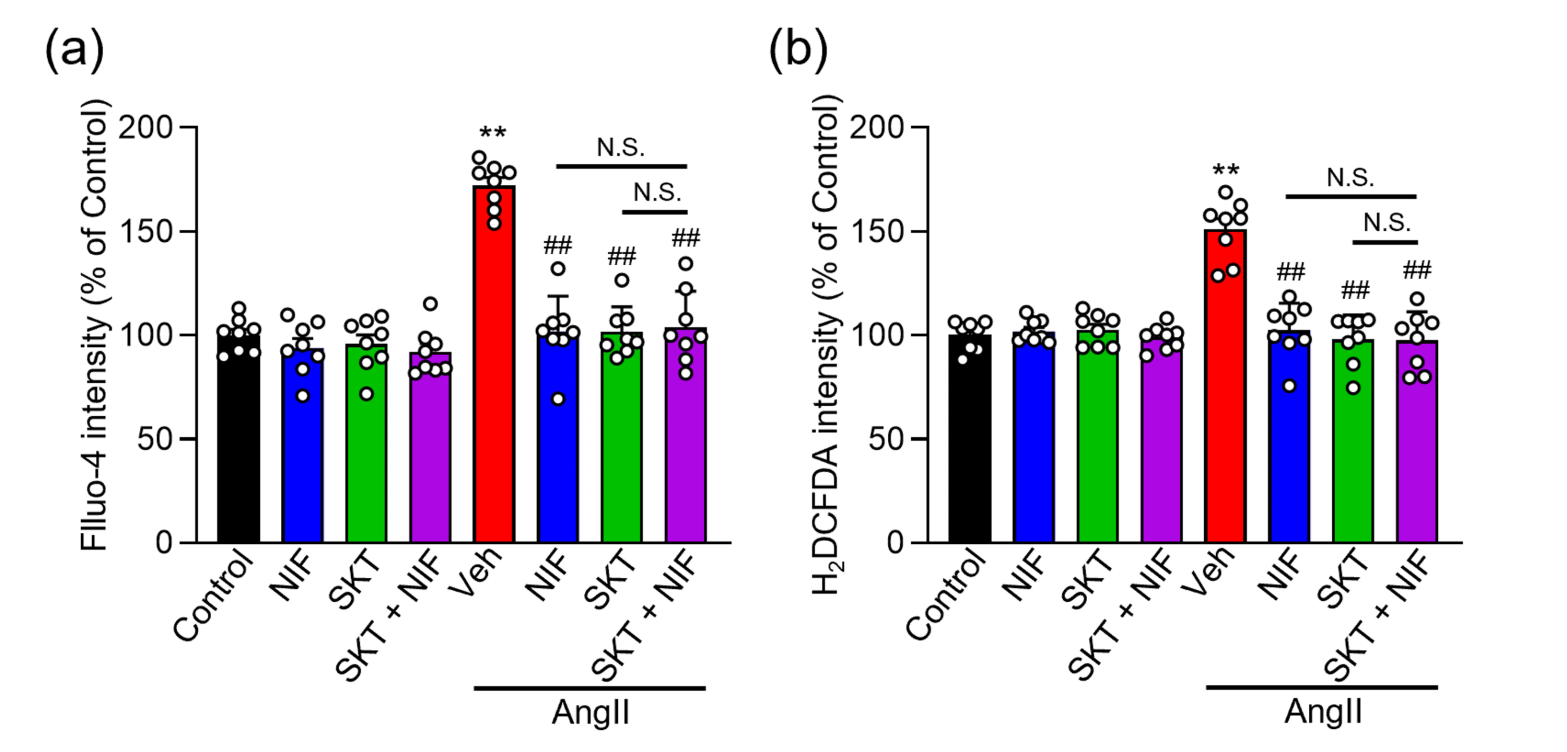
Impact of SKT on intracellular Ca2+ and ROS levels during AngⅡ-induced cardiomyocyte hypertrophy in the presence of nifedipine (a, b) The effects of 250 μg/ml SKT, 1 μM NIF, or 1 μM NIF and 250 μg/ml SKT, or vehicle (Control or Veh) in NRVMs with and without AngⅡ induction of cardiomyocyte hypertrophy for 48 h was assessed by observing intracellular Ca^2+^ concentration (Fluo-4 intensity) (n = 8) (a) and ROS production (H2DCFDA intensity) (n = 8-9) (b). Each column represents the mean ± SEM. **, p < 0.01 indicates a significant difference compared to Control group. ##, p < 0.01 indicates a significant difference compared to AngⅡ + Veh group. N.S. indicates no significant difference. SKT: Shakuyaku-kanzo-to, AngⅡ: angiotensin Ⅱ, ROS: reactive oxygen species, SEM: standard error of the mean

Moreover, treatment with SKT, or SKT and NIF, effectively suppressed the AngⅡ-induced increase in [Ca^2+^]_i_ and ROS production, with no significant differences noted in their inhibitory effects (Figure 5).

These results indicate that SKT inhibits the influx of Ca^2+^ through L-Ca^2+^, thereby suppressing the increase in intracellular Ca^2+^ levels and ROS production, contributing to the mitigation of AngⅡ-induced cardiomyocyte hypertrophy.

## Discussion

This study aimed to investigate the impact of SKT on cardiomyocytes, with a primary focus on its potential to mitigate AngⅡ-induced hypertrophy and cell death. Our findings reveal, for the first time, that SKT exerts a direct effect on cardiomyocytes, effectively preventing AngⅡ-induced hypertrophy and subsequent cell death. In addition, SKT demonstrates protective properties against AngⅡ-induced disruption of intracellular Ca²⁺ homeostasis and ROS production. This is partly achieved by inhibiting Ca²⁺ influx through L-type Ca²⁺ channels, thereby contributing to its cardioprotective effects. Although the precise molecular mechanisms remain to be elucidated, our results suggest that SKT mitigates cardiomyocyte hypertrophy and injury by targeting the aberrant Ca²⁺ and ROS signaling pathways triggered by AngⅡ.

Clinical observations suggest that SKT may precipitate cardiac arrhythmias owing to alterations in serum potassium levels [35]. This underscores the necessity for vigilant monitoring of potassium concentrations during its administration. Notwithstanding this precaution, the primary conclusion of our study is that SKT significantly mitigates AngII-induced cardiomyocyte hypertrophy and injury, thereby demonstrating a direct protective effect on NRVMs. Indeed, clinical observations indicate that the administration of SKT contributes positively to improved mortality rates in patients suffering from diabetic cardiomyopathy [25].

SKT is a traditional Japanese herbal medicine formulation that combines licorice (*Glycyrrhizae Radix*) and peony (*Paeoniae Radix*) in a 1:1 ratio. Glycyrrhizic acid (GA) derived from licorice and peonyflorin (PF) from peony are recognized as its active components, both possessing cardioprotective properties. The inhibitory effect of SKT does not acutely target the L-type Ca²⁺ channel pore, but Ca²⁺ imaging analysis shows a decrease in cytoplasmic Ca²⁺ concentration [36]. This suggests a potential long-term inactivation mechanism involving internalization of L-type Ca²⁺ channels in cardiomyocytes [37]. In addition, there may be an indirect inhibitory effect mediated by sodium-calcium exchanger (NCX), calcium pumps, and background calcium leak [38]. Further studies are required to confirm these possible mechanisms.

GA, a triterpenoid saponin found in licorice roots, is known to influence metabolic regulation. The cardioprotective effects of GA and its aglycones have been documented in various models [39], including pressure overload-induced cardiac hypertrophy and isoproterenol-induced ischemic injury [40,41]. Supportive evidence from studies conducted by Yasuda and Kanzaki indicates that highly lipophilic components from licorice, including GA, exhibit inhibitory effects on smooth muscle contraction in uterine tissues [22,23,26]. The L-type Ca²⁺ channel appears to be a critical route involved in this process [42]. In our study, we utilized cultured cardiomyocytes to induce hypertrophy via AngⅡ stimulation, which triggers Ca²⁺ influx through L-type Ca²⁺ channels [31]. Notably, our combination index results indicated that SKT mitigated the effects of AngⅡ stimulation in NRVMs by counteracting the inhibitory effects of NIF, a selective inhibitor of L-type Ca²⁺ channels (Figure 4e). Furthermore, SKT nearly completely inhibited AngⅡ-induced cardiac hypertrophy, whereas NIF provided only partial inhibition (Figures 1d, 4d). These findings robustly suggest that SKT confers its cardioprotective effects through multiple mechanisms.

PF, a constituent of peonies, has garnered attention for its cardioprotective effects. It has been shown to protect against various heart diseases by suppressing ROS production. For example, PF protects against myocardial ischemia and reperfusion injuries in rats [43-49], heart failure in lipopolysaccharides (LPS)- or cecal ligation and puncture (CLP)-induced sepsis in mice and rats, and cardiac injury in response to coronary artery ligation-induced pressure overload in mice and rats [50-55]. It has been proposed that the protective effects of PF operate through the suppression of inflammatory cytokine production by inhibiting ROS and affecting the inducible nitric oxide synthase (iNOS) signaling pathway [53,55,56]. In models of diabetes-induced myocardial infarction and isoproterenol-induced chronic heart failure, PF appears to protect the heart through mechanisms that do not involve regulating Ca²⁺ influx. Rather, it downregulates the TRPV1/CaMK/CREB/CGRP and TGF-β1/Smad signaling pathways, providing protection against myocardial infarction and fibrosis [57-59]. In addition, PF pretreatment in H9c2 cells has been observed to inhibit apoptosis induced by doxorubicin, acrolein, and AngⅡ by reducing ROS production [60-62]. These findings indicate that PF might engage in protective mechanisms against heart disease through multiple pathways, thus sustaining and enhancing cardiac functions. Its mechanisms span from cellular to organ levels, with a primary focus on cardiac protection and reduction of ROS. Our findings demonstrated the suppression of AngⅡ-induced ROS and consequent cell damage (Figures 2, 3c, b), aligning with prior research. It is anticipated that PF's presence in Paeonia contributes to the inhibition of cell death in myocardial hypertrophic cells by reducing ROS levels.

The combined presence of GA and PF in SKT has demonstrated an inhibitory effect on smooth muscle contraction, with an IC_50_ of approximately 440 μg/ml, as evidenced in a study conducted on human uterine tissue [26]. This finding closely aligns with our observations regarding the inhibition of cardiac hypertrophy in cardiomyocytes (Figure 1c).

This study acknowledges several limitations. First, since cultured cardiomyocytes were utilized, further verification through animal models and clinical trials is essential. Moreover, while it has been observed that SKT operates via voltage-gated L-type Ca^2+^ channels, additional research is necessary to investigate its effects on other calcium channel types and calcium-related factors. Furthermore, previous reports indicate that SKT may induce cardiac arrhythmias, necessitating vigilant management of potassium concentrations during clinical application, which represents another limitation to consider. Lastly, although GA and PF are recognized as the primary active ingredients of SKT, a comprehensive analysis is crucial to elucidate the role of other compounds and to ascertain whether the cardioprotective effects result from the specific combinations and quantitative ratios of these compounds.

## Conclusions

This study highlights the potential of SKT as a therapeutic intervention for cardiovascular conditions characterized by cardiomyocyte hypertrophy and apoptosis. SKT demonstrates cardioprotective properties that may involve modulation of calcium channels and downstream signaling pathways, which could be beneficial in mitigating harmful effects associated with AngⅡ-induced cardiac stress. These findings contribute to understanding the pharmacological actions of SKT and underscore its therapeutic promise in managing cardiac hypertrophy and cell death.
